# Diarrhea accompanies intestinal inflammation and intestinal mucosal microbiota dysbiosis during fatigue combined with a high-fat diet

**DOI:** 10.1186/s12866-023-02896-9

**Published:** 2023-05-25

**Authors:** Jing Liu, Bo Qiao, Ying Cai, Zhoujin Tan, Na Deng

**Affiliations:** College of Chinese Medicine, Hunan University of Chinese Medicine, Changsha, 410208 China

**Keywords:** Fatigue, High-fat diet, Intestinal microbiota, Mucosal barrier, Diarrhea, Inflammation

## Abstract

**Objective:**

It was reported fatigue or a high-fat diet triggers diarrhea, and intestinal microbiota may play central roles in diarrhea. Therefore, we investigated the association between the intestinal mucosal microbiota and the intestinal mucosal barrier from fatigue combined with a high-fat diet.

**Method:**

This study divided the Specific pathogen-free (SPF) male mice into the normal group (MCN) and the standing united lard group (MSLD). The MSLD group stood on water environment platform box for 4 h/day for 14 days, and 0.4 mL lard was gavaged from day 8, twice daily for 7 days.

**Result:**

After 14 days, Mice in the MSLD group showed diarrhea symptoms. The pathological analysis showed structural damage to the small intestine in the MSLD group, with an increasing trend of interleukin-6 (IL-6) and IL-17, and inflammation accompanied by structural damage to the intestine. Fatigue combined with a high-fat diet considerably decreased *Limosilactobacillus vaginalis* and *Limosilactobacillus reuteri*, and among them, *Limosilactobacillus reuteri* positively associated with Muc2 and negatively with IL-6.

**Conclusion:**

The interactions between *Limosilactobacillus reuteri* and intestinal inflammation might be involved in the process of intestinal mucosal barrier impairment in fatigue combined with high-fat diet-induced diarrhea.

## Introduction

Diarrhea is defined by the World Health Organization as excretion three or more times a day, with no fecal shape, and as a thin/watery stool [1, 2]. With the change in people's lifestyles and diets, the number of diarrheal diseases has been increasing year by year and has become a major health problem worldwide [3]. There is no consensus on the specific pathogenesis of diarrhea, which may be related to genetic susceptibility, epithelial barrier defects, immune response disorders, and environmental factors [4, 5]. The dietary composition was found to influence the incidence and progression of diarrhea [6, 7]. Protein and a high-fat diet were associated with diarrhea, significantly reduced *Lactobacillus* and *Bifidobacterium*, and decreased mouse digestive enzyme activity and microbial activity [8, 9]. Lard is a common edible oil used by chinese residents and decreased intestinal microbial diversity in mice fed lard [10]. Decreased intestinal digestive enzyme activity, decreased the number of *Bifidobacterium* and *Lactobacillus* in the intestines, and disrupted glycolipid metabolism in mice fed lard for a long time [11, 12]. Physical activity regulates intestinal microbiota and affects health. The body is unable to provide or maintain the energy load required for prolonged or intense exercise, resulting in performance degradation and fatigue [13]. Excessive exercise reduces microbial diversity and intestinal permeability, damages the intestinal mucosal barrier, increases inflammation, and occurs abdominal pain, and diarrhea, while probiotic therapy can reduce the incidence and severity of gastrointestinal (GI) symptoms [14].

Diarrhea is closely related to intestinal microbiota disorder and intestinal mucosal barrier injury. Diarrheal mice had decreased intestinal microbiota diversity, increased inflammatory factors, decreased secretive immunoglobulin A (sIgA), abnormal energy metabolism, increased harmful intestinal bacteria, and decreased beneficial bacteria [15, 16]. Impairment of intestinal mucosa integrity increased inflammatory factors and destruction of the intestinal mucosal barrier in diarrhea patients [17]. The intestinal mucosal barrier consists of biological, chemical, mechanical, and immune barriers. Among them, intestinal microbiota forms a biological barrier to the intestinal mucosa, intestinal mucosa tissue forms a mechanical barrier, mucus secreted by intestinal mucosa cells forms a chemical barrier, and intestinal mucosa lymphatic tissue forms an immune barrier with immune cells and secretions [18, 19]. Changes in intestinal mucosal permeability and damage to the intestinal mucosal mechanical barrier were found to promote intestinal inflammation leading to diarrhea [20].

Influenced by diet, environment, genetics, drugs, age, etc., human intestinal microbiota has nutritional functions, participates in energy metabolism, maintains the integrity of intestinal mucosa, and regulates immune response, which are important factors in maintaining human health [21, 22]; Related to GI diseases, immune and metabolic diseases, neurological and psychiatric disorders [23]. sIgA is the most secreted immunoglobulin in the intestine and the primary line of defense against pathogen adhesion and colonization in the intestinal mucosa. Goblet cells secrete mucus that forms the intestinal mucus layer, of which Mucin 2 (Muc2) is the core mucin and a major component of the intestinal mucus barrier [24, 25]. Cytokines are small molecule proteins secreted by cells that control cell proliferation and differentiation, regulate angiogenesis, and immune and inflammatory responses, and primarily play a role in the differentiation and activation of immune cells [26]. Interleukin-17 (IL-17) and interleukin-6 (IL-6) are cytokines with many activities. IL-17 stimulates the production of multiple cytokines, such as IL-1β, IL-6, tumor necrosis factor (TNF) -α, and TGF-β, which cause and exacerbate inflammation and play an important role in inflammation, immunity, and autoimmunity [27]. The intestinal mucosal mechanical barrier is the most important part of the intestinal mucosal barrier. The intestinal mucosa acts as a mechanical barrier to protect the intestinal tissue while facilitating the transport of nutrients, water, and waste, and regulating the interaction between the intestinal microbiota and the immune system [28]. The intestinal microbiota has a protective effect on host intestinal epithelial cells and can strengthen the intestinal mechanical barrier. Conversely, intestinal microbiota disruption leads to increased intestinal permeability and damage to the intestinal mechanical barrier [24, 29, 30].

Therefore, we established a diarrhea model in mice induced by fatigue combined with a high-fat diet to detect Muc2, sIgA, IL-6, and IL-17, analyze intestinal mucosal microbiota, and observe small intestinal pathology. This study aims to analyze the characteristics of intestinal mucosal microbiota in diarrhea, investigate the relationship between characteristic microbiota and mucosal barrier index, and investigate the role of the mucosal barrier in diarrhea caused by fatigue combined with a high-fat diet.

## Materials and methods

### Animal

To rule out the effect of sex on intestinal microbiota [31], specific pathogen-free (SPF) male Kunming mice (20 ± 2 g, license: SCXK (Hunan) 2019–0004) were purchased from Hunan Slx Jingda Experimental Animal Co (license: SYXK (Hunan) 2019–0009). The animals were bred in the laboratory animal center of the Hunan University of Chinese Medicine, with a temperature of 23–25 ℃, a humidity of 47–53%, a free diet, and drinking water during adaptive feeding.

### Diet

Mice fed Co60 irradiated experimental mice growth and reproduction feed with a composition detailed in Table 1. It is supplied by the Animal Experiment Center of Hunan University of Chinese Medicine and produced by Jiangsu Medison Biomedical Co. Jinluo refined lard is mainly composed of energy (44%) and fat (167%) and is produced by XinCheng Jinluo Meat Products Co (Production license: SC10337130200099; Production Lot No: GB 10146.) It is stored at room temperature and gavaged at 37 °C.

**Table 1:** Common feed (per kg of feed)

Component	Content
water (g)	≤ 100
crude protein (g)	≥ 200
crude fiber (g)	≥ 40
crude fat (g)	≤ 50
crude ash (g)	≤ 80
calcium (g)	10–18
phosphorus (g)	6–12
calcium: phosphorus	1.2: 1—1.7: 1
lysine (g)	≥ 13.2
methionine and cysteine (g)	≥ 7.8

### Animal grouping and intervention

**Fig. 1:**
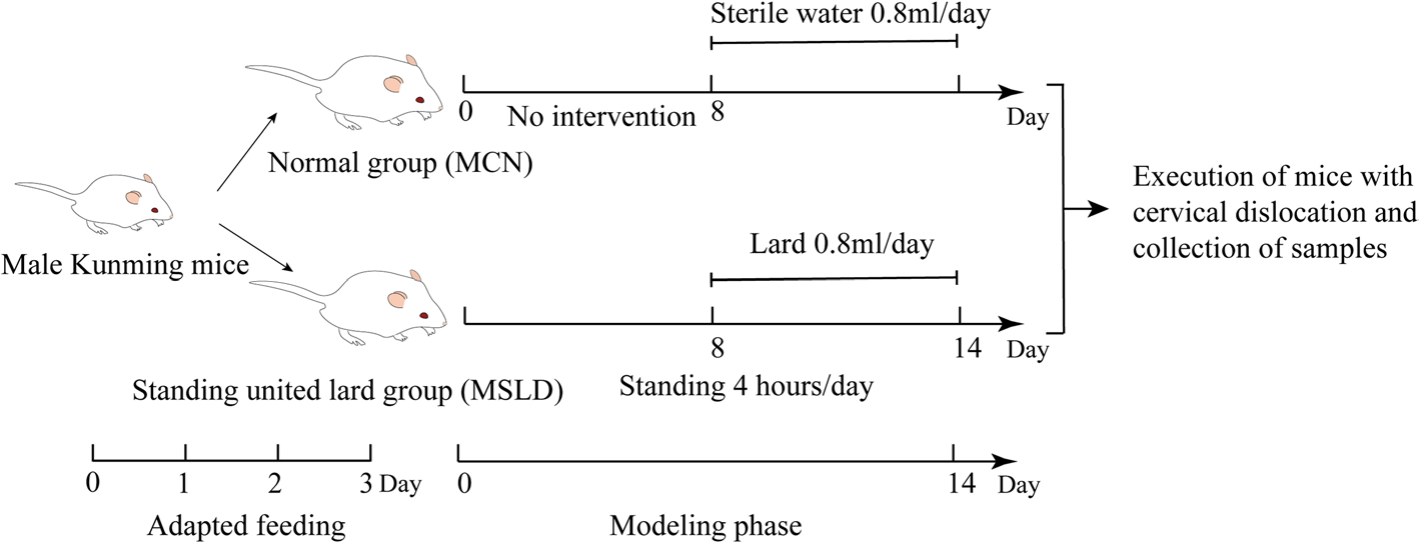
Experimental design and general conditions of the animals

After 3 days of adaptive feeding, the 20 male Kunming mice were randomly divided into the control group (MCN) and the standing united lard group (MSLD). The MCN group was not treated with intervention for 7 days and was given 0.4 mL of sterile water daily starting on day 8. Based on the literature [32–36] and pre-experimental results [37, 38], fatigue combined with a high-fat diet was used to induce diarrhea in mice. The MSLD group stand on a small homemade water environment platform box for 4 h/day for 14 days, and 0.4 mL lard gavaged from day 8, twice daily for 7 days. All the animal experiments were carried out by the animal control and use committee approved by the Hunan University of Chinese Medicine (Ethical approval number: LL2022062308). Figure 1 shows the experimental design and the specific experimental procedure.

### General features

The animals were observed daily in the morning, observing their body size, fecal shape, eyes, hair, and activity, recording their initial weight, and then weighing them every other day. The mice's initial feces were recorded and then collected daily from 9: 00 a.m. to 9: 30 a.m., the number of feces in each group was recorded to observe the texture of the feces, and photos were taken of the feces.

### Organ index

An experienced experimenter quickly executed mice using cervical dislocation at the end of the experiment. The spleen, thymus, and liver were immediately dissected and removed, and the blood was drained with filter paper and weighed. The calculation of the organ index was done by using the formula: Organ index = organ weight (mg)/body weight (g).

### Detection of sIgA in serum

After 14 days of intervention, blood was collected by eyeball, stood at 4 °C for 1–2 h, centrifuged at 3000 r for 15 min and the upper serum was collected. Enzyme-linked immunosorbent assays (ELISA) were performed according to the kit instructions, followed by an enzyme labeling analyzer to detect sIgA levels in serum samples (the kit was provided by Quanzhou Konodi Biotech Ltd.).

### Detection of Muc2, sIgA, IL-6 and IL-17 in small intestinal tissue

Under sterile conditions, small intestinal tissues were collected after rinsing the contents of the small intestine with saline. According to the ELISA assay instructions, a certain amount of small intestine was mixed with saline in a ratio of 1: 9 and ground in a high-speed centrifuge at 4 °C for 3 min. The tissue homogenate was centrifuged for 15 min and the supernatant was absorbed. ELISA was performed according to the kit instructions, followed by an enzyme labeling analyzer to detect Muc2, sIgA, IL-6, and IL-17 in small intestine tissue (the kit was provided by Quanzhou Konodi Biotech Ltd.).

### Histopathology of the small intestine

Under sterile conditions, small intestinal tissues were collected and fixed in a 4% paraformaldehyde solution at room temperature after rinsing the contents of the small intestine with saline. According to dehydration of gradient ethanol, xylene transparent paraffin was embedded in four um sections, routinely dewaxed, then stained with hematoxylin and eosin-methylene blue solution (hematoxylin–eosin staining, HE), and sealed with neutral gum.

### Collection of intestinal mucosa samples

Intestinal mucosa samples were collected concerning previous methods [39]. In sterile conditions, intestinal tissue from the pyloric to the ileocecal region was cut lengthwise with sterile scissors, the contents of the intestine were flushed with sterile saline, and the intestinal mucosa of each mouse was individually scraped with sterile lids. The mucosa was collected in an EP tube and stored at -80 ℃.

### DNA extraction、16S rRNA gene amplicon sequencing and sequence analysis

All samples were sent to Shanghai Paceno Biotech Co., Ltd. (Shanghai, China) for processing. The total microbial genomic DNA of each tube of samples was extracted following the steps of the OMEGA Soil DNA Kit (M5635-02) kit to extract nucleic acid instructions. The quantity and quality of extracted DNAs were measured using a NanoDrop NC2000 spectrophotometer (Thermo Fisher Scientific, Waltham, MA, USA) and agarose gel electrophoresis, respectively. Forward primer 27F (5′-AGAGTTTGATCMTGGCTCAG-3′) and reverse primer 1492R (5′-ACCTTGTTACGACTT-3′) were used for PCR amplification of bacterial 16S rRNA near the full-length gene. The 16S rRNA gene was amplified by polymerase chain reaction (PCR) using Q5 high-fidelity DNA polymerase. PCR products were detected by 2% agarose gel electrophoresis and purified by a DNA gel extraction kit. The recovered PCR amplification products were quantified by fluorescence intensity using the dsDNA Assay Kit. Based on the fluorescence quantification results, the samples were mixed proportionally according to the sequencing requirements of each sample. The intestinal mucosal microbiota sequencing data has been uploaded to the NCBI database: PRJNA903506 (https://www.ncbi.nlm.nih.gov/).

### Bioinformatics

Sequence data analyses were mainly performed using QIIME2 and R packages (v3.2.0). ASV-level alpha diversity indices, such as the Chao1 richness estimator, Observed species, Shannon diversity index, and Simpson index were calculated using the ASV table in QIIME2. ASV-level ranked abundance curves were generated to compare the richness and evenness of ASVs among samples. Beta diversity analysis was performed to investigate the structural variation of microbial communities across samples using Bray–Curtis metrics (Bray and Curtis, 1957) and visualized via principal coordinate analysis (PCoA) and nonmetric multidimensional scaling (NMDS). A Venn diagram was generated to visualize the shared and unique ASVs among samples or groups using the R package “VennDiagram”, based on the occurrence of ASVs across samples/groups regardless of their relative abundance. LEfSe (Linear discriminant analysis effect size) was performed to detect differentially abundant taxa across groups using the default parameters. Random forest analysis was applied to discriminate the samples from different groups using QIIME2 with default settings.

### Statistical analysis

Statistical analysis was performed using SPSS 25.00 software, and each group of data was expressed as mean ± standard deviation. If the two sets of data conform to normal distribution and homoscedasticity, the independent sample t-test is used, and the non-homoscedasticity T-test is used. Mann–Whitney U assays were used if the data did not match the normal distribution and the non-homoscedasticity (*p* < 0.05 indicated statistical difference).

## Result

### General characteristics of mice with fatigue combined with a high-fat diet

**Fig. 2:**
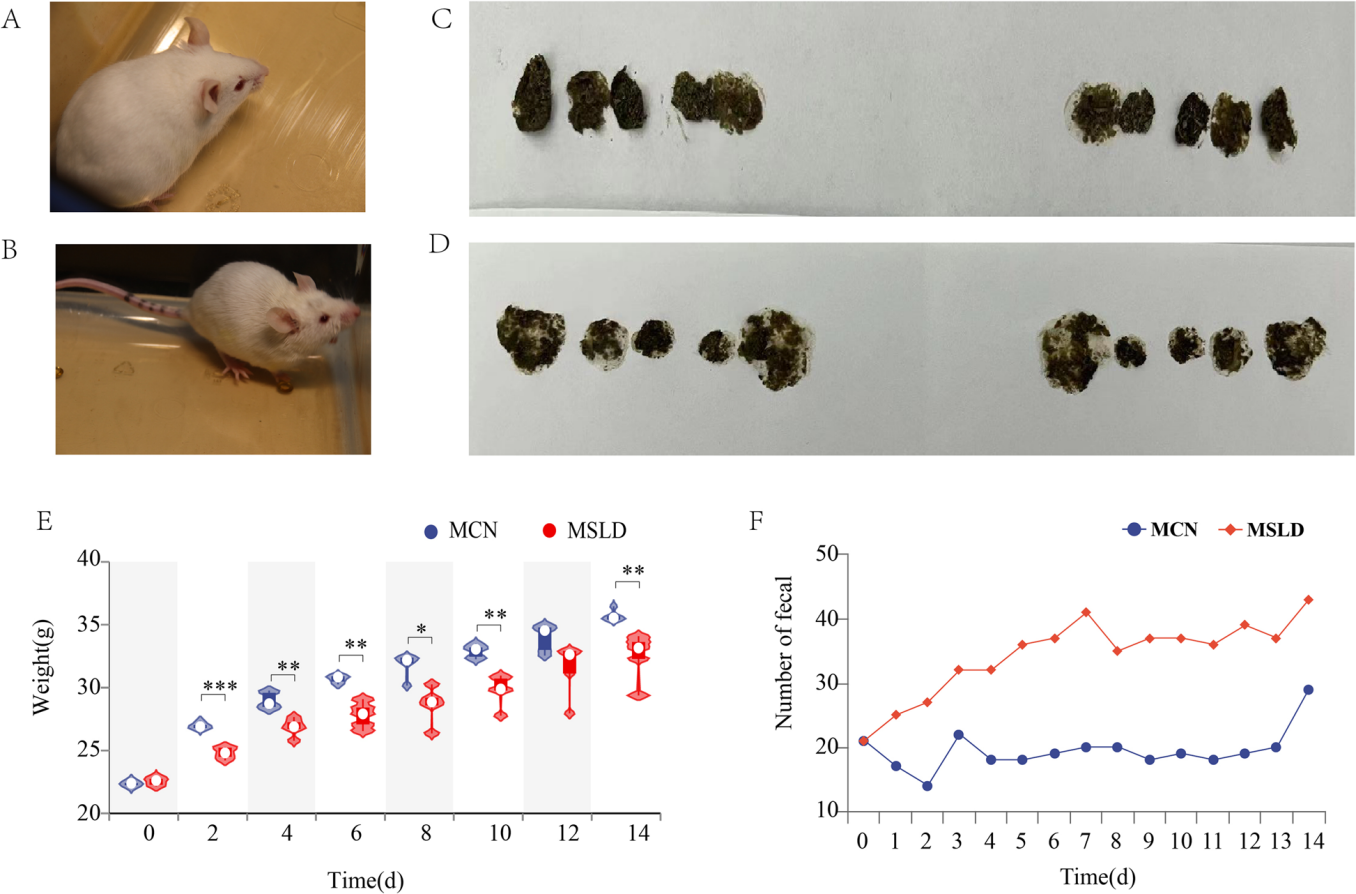
**A** General characteristics of the MCN group after 14 days. **B** General characteristics of the MSLD group after 14 days. **C** Fecal images of the MCN group after 14 days, **D** Fecal images of the MSLD group after 14 days, **E** Violin chart of the weight difference in the MCN and MSLD groups (*n* = 5). **F** Line graph of the number of fecal in half an hour for the MCN and MSLD groups (*n* = 5 (*p < 0.05, **p < 0.01, ***p < 0.001))

During adaptive feeding, the mice were responsive, with flexible eyes, glossy hair, ruddy skin mucosa, long strips of feces, close body weight, and several fecal movements. After 14 days, The MCN group had the same status as before (Fig. 2A and C). The MSLD group showed a marked decrease in activity, frequent squinting, matte and slightly yellow skin, pale mucosa, and soft and shapeless feces (Fig. 2B and D). Compared to the MCN group, the MSLD group had an increased number of fecal and decreased body weight (*p* < 0.05, Fig. 2B, D, E, and F).

### Organ indices, Muc2, and sIgA of mice with fatigue combined with a high-fat diet

As shown in Table 2, compared to the MCN group, the spleen index was lower, and thymus and liver indices were higher in the MSLD group (*p* > 0.05). Fatigue combined with a high-fat diet had little effect on organ function in mice.

**Table 2:** Organ indices (Organ indices = Organ weight/Mouse weight, mean ± standard deviation)

Organ indices	MCN	MSLD
spleen(mg/g)	2.98 ± 0.45	2.86 ± 0.25
thymus(mg/g)	2.53 ± 0.77	3.24 ± 0.56
liver(mg/g)	53.7 ± 8.28	58.09 ± 5.99

**Fig. 3:**
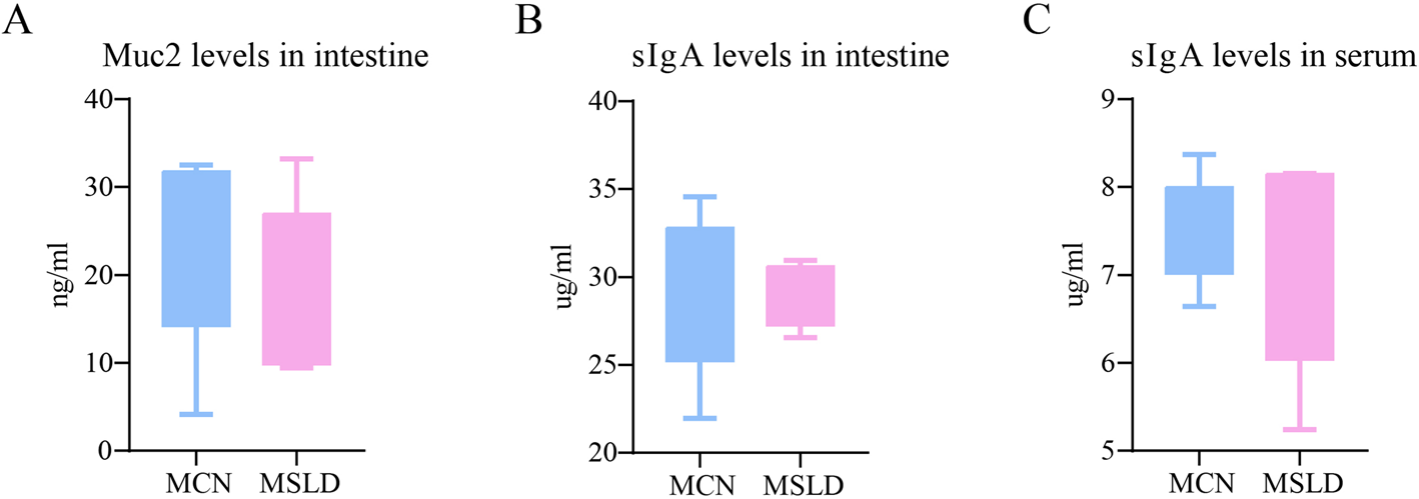
**A** Muc2 levels in intestinal tissue; **B** sIgA levels in intestinal tissue; **B** sIgA levels in serum

Muc2 in small intestine tissue forms a chemical barrier to the intestinal mucosa, sIgA forms an immune barrier to the intestinal mucosa, and sIgA in serum reflects overall immune levels. As shown in Fig. 3A-C, compared to the MCN group, the MSLD group presented an increased trend of IL-6 and IL-17 and a decreased tendency of sIgA and Muc2 (*p* > 0.05).

### Small intestine tissue morphology of mice with fatigue combined with a high-fat diet

**Fig. 4:**
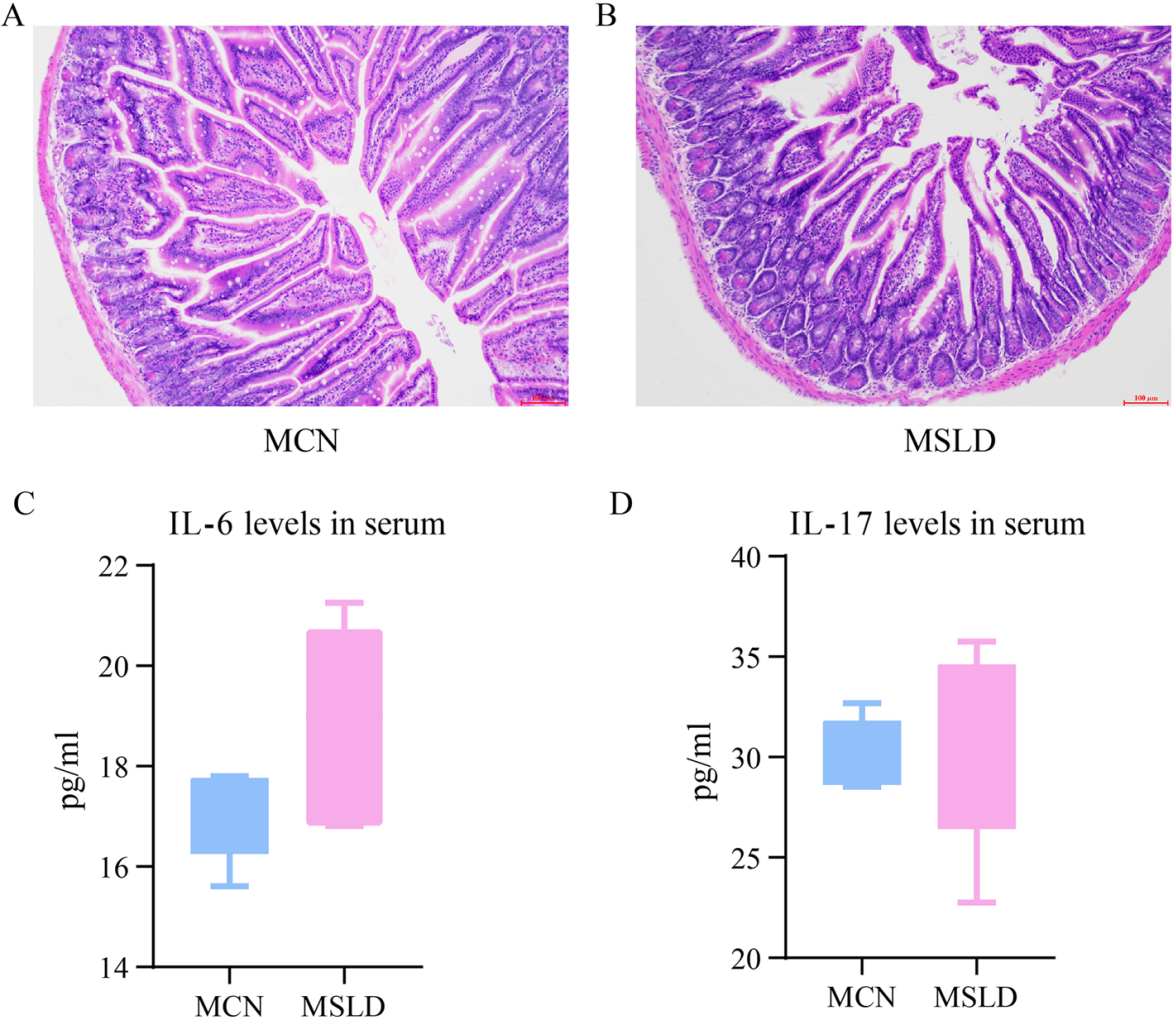
**A** The HE dye of small intestine tissue from the MCN group (100X). **B** The HE dye of small intestine tissue from the MSLD group (100X). **C** IL-6 levels in intestinal tissue; **D** IL-17 levels in intestinal tissue

In the MCN group, the mucosa of the small intestine is clear, the layer is complete, the muscularis mucosae is intact, there is no obvious edema, inflammation, or lymphocyte infiltration, and it is a normal tissue structure (Fig. 4A). The MSLD group had a clear mucosal structure, disrupted intestinal villi continuity, thinning muscularis mucosae, atrophy of the small intestine gland, and infiltration of lymphocytes (Fig. 4B). Compared to the MCN group, IL-6, and IL-17 were higher in the MSLD group (*p* > 0.05, Fig. 4C to D).

### Intestinal mucosal microbiota of mice with fatigue combined with a high-fat diet

#### Effects of fatigue combined with a high-fat diet on the number and diversity of ASV in mouse intestinal microbiota

**Fig. 5:**
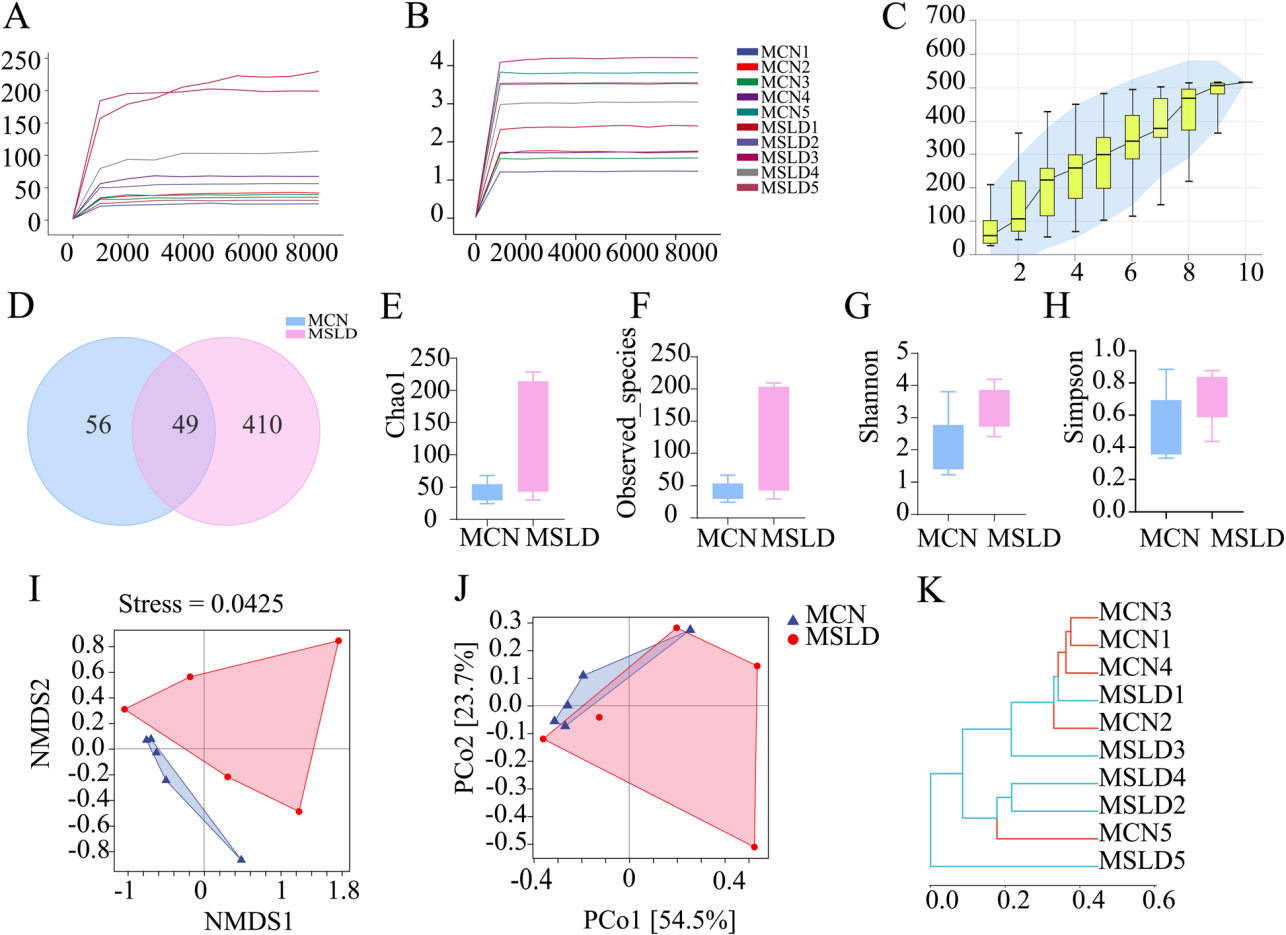
**A** Chao1 Wiener curves of intestinal mucosal microbiota. **B** Shannon Wiener curves of intestinal mucosal microbiota. **C** Species accumulation curves. **D** Venn diagram: distribution of the number of ASV of intestinal mucosal microbiota. **E** Chao1 index. **F** Observed species index. **G** Shannon index. **H** Simpson index. **I** PCoA analysis. **J** NMDS analysis. **K** Clustering analysis

The increased number of ASV decreased with the increase in the number of sequenced data, and the curve flattened, suggesting that the amount of sequenced data was sufficient for this analysis (Fig. 5A and B). The depth of sequencing in this experiment is sufficient to reflect the microbial diversity contained in the community sample, the reasonableness of the experimental design, and the reliability of the data (Fig. 5C).

Combining sequences with similarities of more than 100% into one ASV cluster, the analysis showed 105 ASV in the MCN group, and 459 ASV in the MSLD group (Fig. 5D). These results suggest that fatigue combined with a high-fat diet may increase the number of ASV. Alpha diversity analysis reflects the abundance and diversity of the microbiota. The Chao1, Observed species indices measure the number of species in a community, and the larger the index, the more species there are. Shannon and Simpson’s indices are used to measure species diversity, primarily the number and uniformity of species. The higher the Shannon index, the more diverse Alpha is; the higher the Simpson index, the lower the diversity of Alpha. The Chao1 observed species, Shannon, and Simpson indices were increased in the MSLD group (*p* > 0.05; Fig. 5E-H).

NMDS reflects the information of the distance matrix between samples. The MCN group was concentrated and the MSLD group was widely distributed (Fig. 5I). PCoA is used to study similarities or differences in the composition of a sample community, where two samples are closer together, representing a more similar composition of the two species. When PCo1 is 54.5%, and PCo2 is 23.7%, the MCN and MSLD groups are far apart and have large compositional differences (Fig. 5J). Cluster analysis showed that the MCN and MSLD groups could cluster together better, with small intra-group differences and large inter-group differences between the two groups (Fig. 5K).

#### Effects of fatigue combined with a high-fat diet on intestinal mucosal microbiota composition in mice

**Fig. 6:**
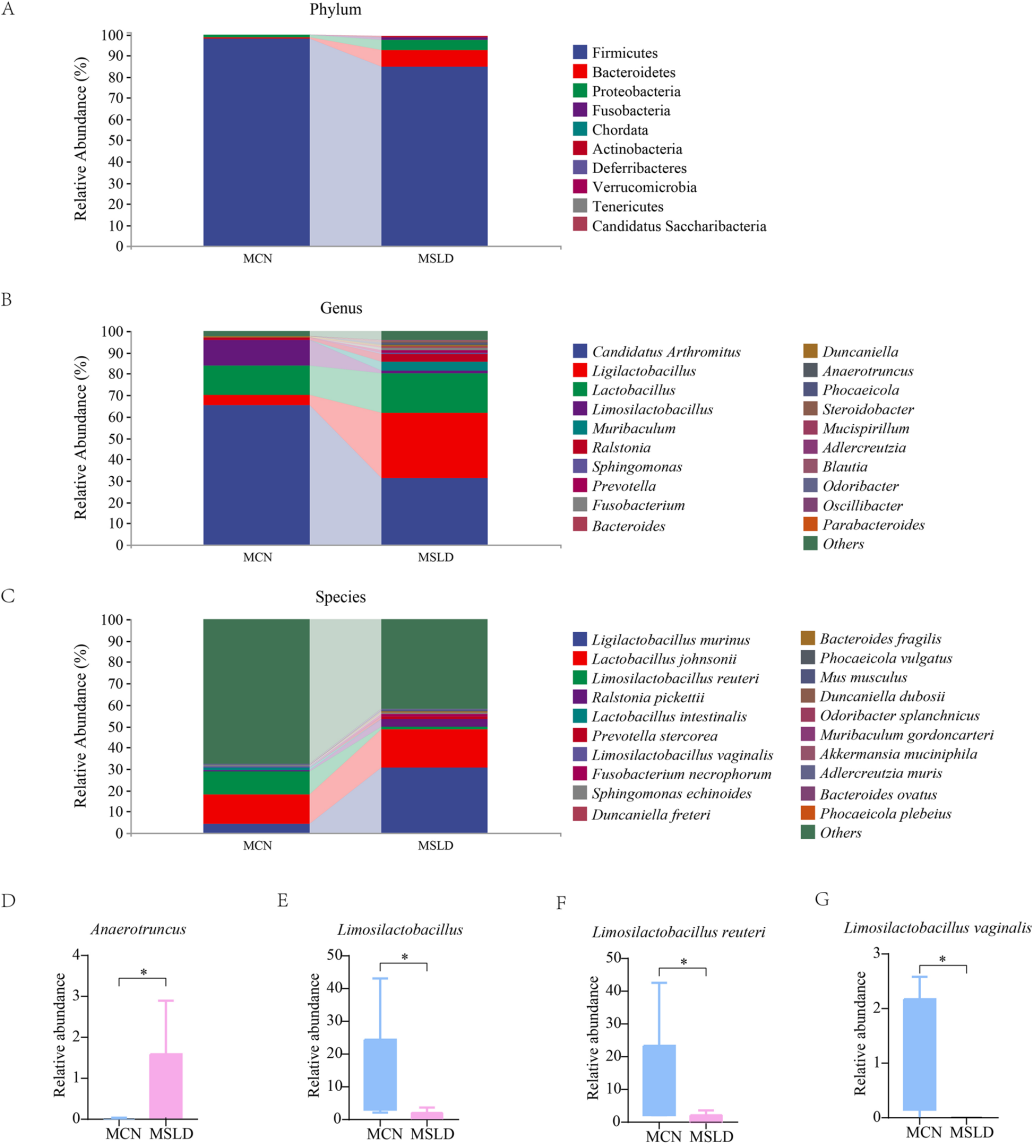
**A** phylum level intestinal mucosa microbiota. **B** genus level intestinal mucosa microbiota. **C** species level intestinal mucosa microbiota. **D**-**G** Genus and species of intestinal mucosa dominant bacteria in the MCN and MSLD groups (*p < 0.05)

Figure 6A shows the relative abundance of intestinal mucosal microbiota at the phylum level. The MCN group had the largest proportion of Firmicutes, followed by Bacteroidetes, and Proteobacteria. However, Bacteroidetes and Proteobacteria increased and Firmicute and Firmicute/Bacteroidetes decreased in the MSLD group (*p* > 0.05). Figure 6B shows the relative abundance of intestinal mucosa microbiota at the genus level, *Candidatus arthromitus* was the first dominant genus which had 75.23% intestinal mucosal in the MCN group and 30.94% in the MSLD group. Compared to the MCN group, *Limosilactobacillus* was significantly reduced in the MSLD group (*p* < 0.05), and *Anaerotruncus* was significantly increased (*p* < 0.05, Fig. 6D to E). Figure 6C shows the relative abundance of intestinal mucosal microbiota at the species level, with *Ligilactobacillus murinus* (4.57%), *Lactobacillus johnsonii* (13.48%), and *Limosilactobacillus reuteri* (10.8%) dominating the MCN group. Compared to the MCN group, *Lactobacillus murinus* (30.63%) and *Lactobacillus johnsonii* (18.12%) increased in the MSLD group (*p* > 0.05), and *Limosilactobacillus reuteri* and *Limosilactobacillus vaginalis* significantly decreased (*p* < 0.05, Fig. 6F to G).

#### Effects of fatigue combined with a high-fat diet on intestinal mucosal characteristic microbiota in mice

**Fig. 7:**
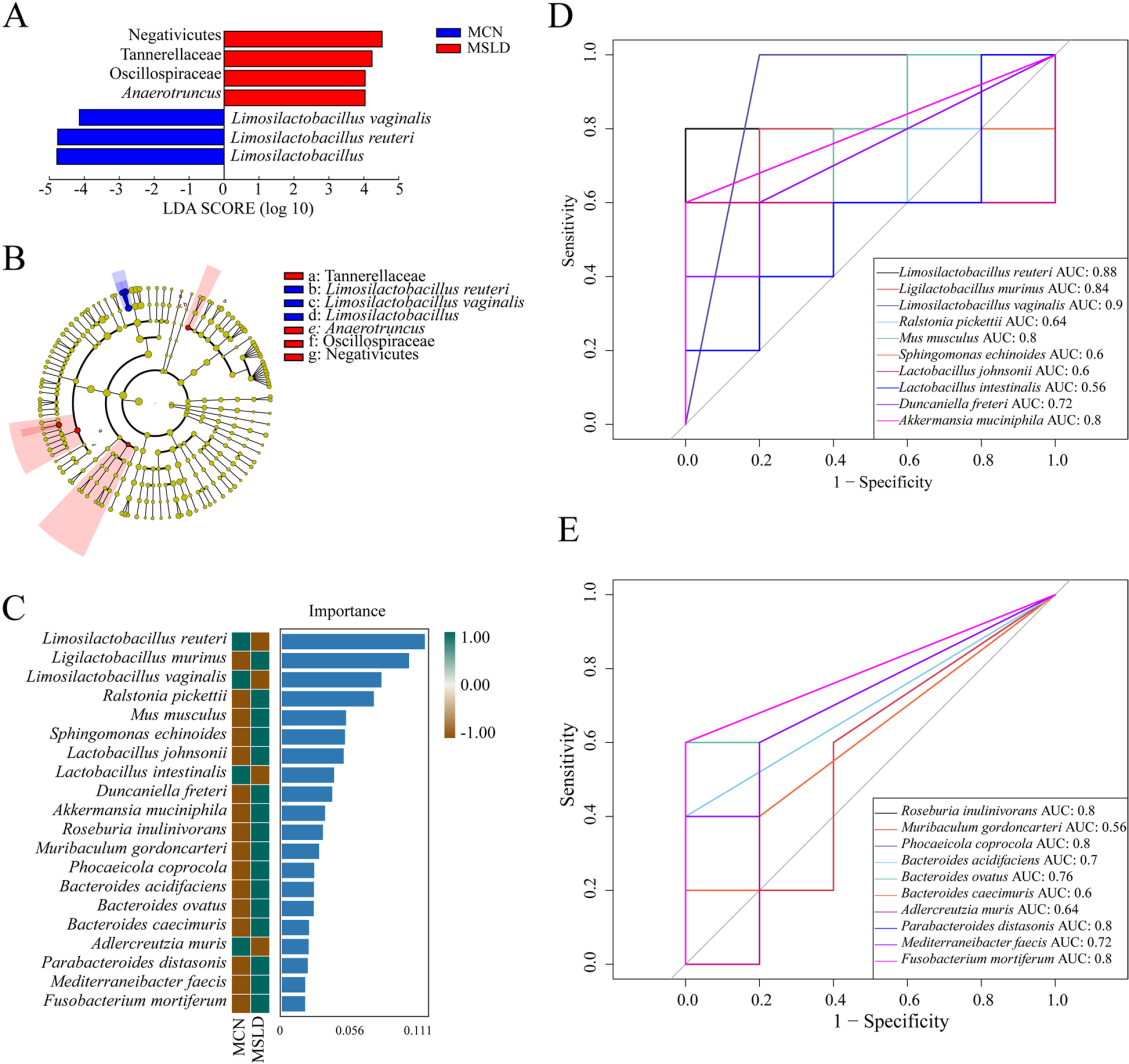
Core characteristic bacterial analysis of intestinal mucosal microbiota. **A** LDA diagram. **B** Cladogram diagram. **C** Random Forest diagram of species level. **D**-**E** ROC curve of species

As shown in Fig. 7A and B, the LEfSe analysis identified differentially altered characteristic microbiota, with LDA scores greater than 4, of which 7 bacteria were identified as key differentiators. Negativicutes, Tannerellaceae, Oscillospiraceae, and *Anaerotruncus* are the characteristic bacteria of the MCN group, *Limosilacillus vaginalis*, *Limosilacillus reutrei*, and *Limosilactobacillus* are the characteristic bacteria of the MSLD group. Combined with a randomized forest diagnostic model (Fig. 7C), the MCN and MSLD groups were distinguished using 20 different species levels of bacteria. ROC curves showing large areas under *Limosilactobacillus reuteri, Ligilactobacillus murinus, Limosilactobacillus vaginalis, Mus musculus, Akkermansia muciniphila, Roseburia inulinivorans, Phocaeicola coprocola, Parabacteroides distasonis*, and *Fusobacterium mortiferum* curves(Fig. 7D and E). Among them, *Limosilactobacillus vaginalis* (AUC = 0.9) showed the highest AUC, suggesting that fatigue combined with a high-fat diet resulted in characteristic enrichment of *Limosilactobacillus vaginalis*, which can be identified as a key bacterium for diarrhea.

#### Effects of fatigue combined with a high-fat diet on intestinal mucosal microbiota function in mice

**Fig. 8:**
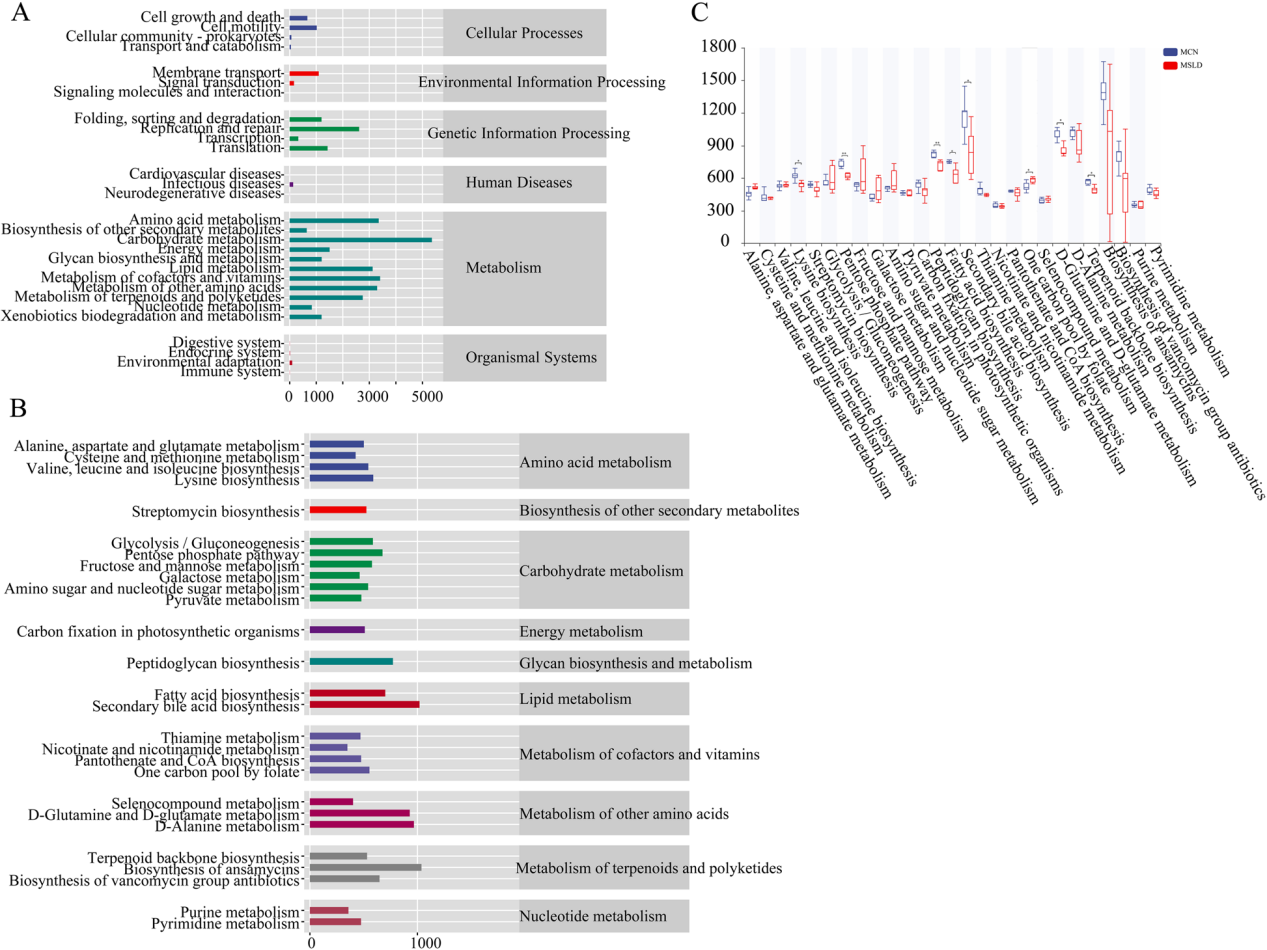
Prediction of intestinal mucosal microbiota metabolism based on PICRUSt2. **A-B** Predicted abundance of KEGG function with horizontal coordinates of KEGG functional pathway and longitudinal coordinates of KEGG functional pathway classification. **C** Comparison between groups of metabolic functional groups (*p < 0.05, ** *p* < 0.01, ***p < 0.001)

To determine the metabolic and functional effects of fatigue combined with a high-fat diet on intestinal mucosal microbiota in mice, PICRUSt2 analysis based on the KEGG database [40–42] predicted microbiota-related metabolic pathways. Figure 8A shows six major functional types (Cellular Processes, Environmental Information Processing, Genetic Information Processing, Human Diseases, Glycan Pathways, and Metabolism) consisting of 29 functional pathways, with the greatest abundance of Metabolism pathways.

The median metabolic function of the Metabolism Level 3 pathway > 342.8465 was selected (27 classes). As shown in Fig. 8B, the main metabolic pathway of Amino acid metabolism, Carbohydrate metabolism, Metabolism of cofactors and vitamins, Metabolism of terpenoids and polyketides, and Lipid metabolism. As shown in Fig. 8C, compared to the MCN group, Lysine biosynthesis, Pentose phosphate pathway, Peptidoglycan biosynthesis, Fatty acid biosynthesis, Secondary bile acid biosynthesis, D-Glutamate metabolism, and Terpenoid backpack biosynthesis were significantly reduced in the MSLD group (*p* < 0.05) and One carbon pool by folate was significantly increased (*p* < 0.05).

### Correlation analysis of sIgA, Muc2, IL-6, IL-17, metabolic pathways and characteristic microbiota

**Fig. 9:**
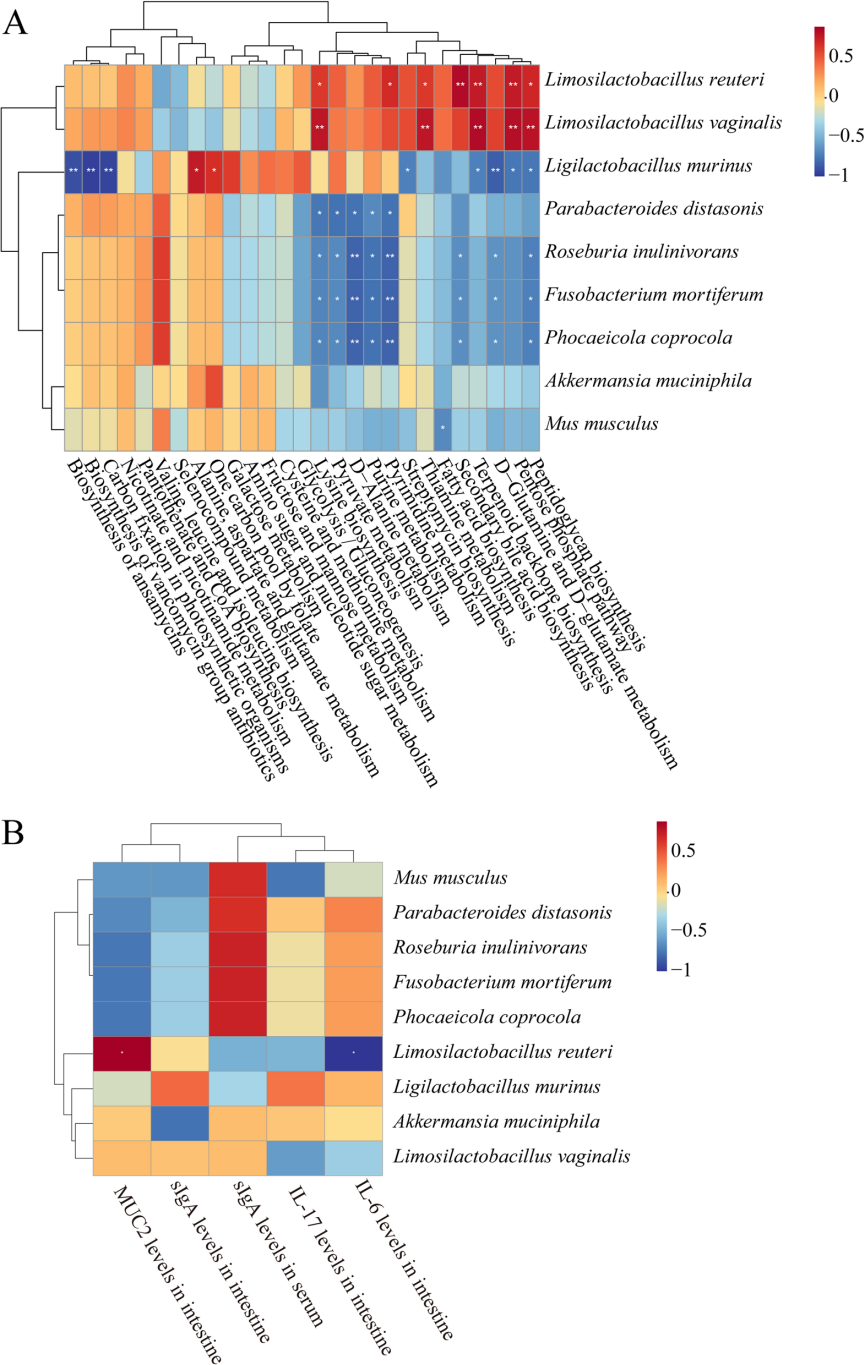
Spearman correlation analysis heatmap: blue represents negative correlation, red represents positive correlation, and the closer the color is to blue, the stronger the negative correlation between the two parameters, and the closer the color is to red, the stronger the positive correlation between the two parameters. **A** Correlation heatmap of intestinal mucosa microbiota and metabolic pathways. **B** Correlation heatmap of intestinal mucosa microbiota with sIgA, Muc2, IL-6, and IL-17

To investigate the relationship between intestinal mucosal microbiota, metabolic pathway, and intestinal mucosal barrier, we performed Spearman correlation analysis of sIgA, Muc2, IL-6, and IL-17 by selecting nine signature enrichment diagnostic differentially enriched bacteria at the species level and the metabolic pathways with abundance in the top 27. The aim is to determine the key role of intestinal mucosal microbiota in maintaining the stability of the intestinal microenvironment. Correlation heat maps (Fig. 9A and B) show that *Limosilactobacillus reuteri* and *Limosilactobacillus vaginalis* are significantly associated with Pentose photosynthesis pathway, Peptidoglycan biosynthesis, Lysine biosynthesis, Terpenoid backbone biosynthesis, Thiamine metabolism. *Limosilactobacillus reuteri* was significantly positively correlated with Muc2 levels in the small intestine and negatively correlated with IL-6.

## Discussion

In recent years, studying the effects of diet and exercise on the body based on intestinal microbiota has become a hot topic. High-fat diets (HFD) promote inflammatory markers such as IL-6, and TNF-α, and increase the risk of liver toxicity, leading to dysfunctional energy metabolism and causing metabolic disease and inflammation [43–45]. Chronic physical fatigue impairs normal body functions, leading to endocrine disruption and decreased immunity [46]. The intestinal barrier may be compromised by severe structural damage to mucous membranes or by changes in the barrier's regulatory composition. Associated cytokines such as IL-6 and IL-17 are involved in intestinal mucosal inflammation, while damage to the intestinal barrier is associated with inflammation of the small intestinal mucosa [47, 48]. Muc2 and sIgA are essential for the intestinal mucosal mucus barrier and immune barrier. Mice with Muc2 deficiency develop colonic inflammation and mucosal hyperplasia [49]. In contrast, sIgA expression is reduced and intestinal barrier function is impaired [50]. In this study, we found that fatigue combined with a high-fat diet led to led to an increased number of feces, shapeless feces, losing weight, and diarrhea symptoms in mice. Further analysis confirmed that fatigue combined with a high-fat diet causes inflammation of the small intestine and disrupts the intestinal mucosal barrier. Thus, fatigue combined with a high-fat diet leads to diarrhea in mice, which may be associated with increased inflammatory factors and decreased immune factors.

Numerous studies have shown that the intestinal microbiota mediates the relationship between diet and health, and influences the onset and progression of disease. HFD alters microbial diversity, leading to intestinal microecological disorders that promote local inflammation and increase intestinal wall permeability [51]. The abundance of Firmicute, Bacteroidetes, and Actinobacteria was associated with host obesity and increased Firmicute/Bacteroidetes ratios and changes in bacterial species were associated with obesity progression [52]. The results of this study showed that fatigue combined with a high-fat diet increased the abundance and diversity of intestinal mucosal microbiota and decreased Firmicute/Bacteroidetes, which explains why mice given gavage with high-fat lard did not gain weight.

*Anaerotruncus* promotes inflammation and tumorigenesis, undermines the integrity of the epithelial barrier, has pro-inflammatory properties, and has been identified as a potential biomarker for colorectal cancer recurrence and patient prognosis [53]. *Lactobacillus* regulates microbiota, attenuates pro-inflammatory cytokines, prevents inflammation, restores barrier function, modulates intestinal microbiota as well as metabolic and immune parameters in obese mice under HFD, and acts as a probiotic strain for the treatment of obesity [54, 55]. *Limosilactobacillus reuteri* reduces inflammatory response, repairs epithelial tissue structure, protects barrier function, and prevents colitis [56]. HFD increases harmful bacteria (*Anaerotruncus)*, reduces the abundance of dominant bacteria and beneficial bacteria such as *Lactobacillus johnsonii* and *Lactobacillus reuteri,* promotes inflammation, and damages the intestinal barrier [57–59]. In this study, fatigue combined with a high-fat diet intervention significantly increased *Anaerotruncus*, while significantly decreasing *Limosilacillus* *,* *Limosilacillus vaginalis*, and *Limosilacillus reutrei*, which may promote inflammation, destroy the intestinal mucosal barrier, and lead to diarrhea in mice. Thus, fatigue combined with a high-fat diet intervenes in mice with reduced harmful and beneficial bacteria, which may be an important cause of diarrhea. Based on LEfSe analysis, random forest diagnosis, and correlation analysis showed that *Limosilactobacillus reuteri* was a different bacterium in the MSLD group, which has a large AUC value and was significantly positively correlated with Muc2 levels in the small intestine and negatively correlated with IL-6. *Limosilactobacillus vaginalis*, whose abundance is associated with Muc2 and IL-6 levels, can be used as a characteristic bacterium for diarrhea diagnosis.

Intestinal homeostasis is determined by complex interactions between the intestinal microbiota, epithelial barrier, and host immune system. Intestinal microbiota participates in synthesizing and metabolizing proteins, carbohydrates, lipids, vitamins, and minerals, balancing salt and water intake, increasing energy in intestinal epithelial cells, and breaking down lipids and cholesterol [60]. By predicting the metagenomic function of the microbiota, we found significant changes in the Metabolism pathway in mice following overexertion combined with lard dietary intervention. Micronutrient deficiency increases the incidence of bowel diseases [61]. Folic acid deficiency can lead to severe carbon metabolism abnormalities and lead to chronic disease and developmental disorders [62]. Lysine is one of the essential amino acids in the human body. It can synthesize proteins, regulate fat metabolism, promote the release of endocrine hormones, and strengthen immunity [63]. Lysine is an important precursor to the synthesis of glutamate, the most important excitable neurotransmitter in the mammalian central nervous system. Excessive lysine is metabolized as a source of energy, and a lack of lysine in the diet will impair animal immunity and increase animal susceptibility to infectious diseases [64]. According to Spearman correlation analysis, *Limosilactobacillus reuteri*, and *Limosilactobacillus vaginalis* were significantly positively associated with the Pentose photosynthesis pathway, Peptidoglycan biosynthesis, Lysine biosynthesis, Terpenoid backbone biosynthesis, and Thiamine metabolism. Therefore, we deduce that metabolic function may be associated with changes in characteristic bacterial interactions following fatigue combined with a high-fat diet, suggesting that microbiota influences metabolic function leading to diarrhea in mice.

## Conclusion

The interactions between *Limosilactobacillus reuteri* and intestinal inflammation might be involved in the process of intestinal mucosal barrier impairment in fatigue combined with high-fat diet-induced diarrhea.

## Data Availability

The data underlying this study was available within the manuscript. The gut content microbiota sequencing data has been uploaded to the NCBI database: PRJNA903506.
